# Knowledge, attitudes and practices of health care professionals towards adverse drug reaction reporting in public sector primary health care facilities in a South African district

**DOI:** 10.1007/s00228-020-02862-8

**Published:** 2020-04-15

**Authors:** H. M. Haines, J. C. Meyer, R. S. Summers, B. B. Godman

**Affiliations:** 1 Tshwane Regional Pharmacy, Tshwane, South Africa; 2 Division of Public Health Pharmacy and Management, School of Pharmacy, Sefako Makgatho Health Sciences University, Molotlegi Street, Ga-Rankuwa, 0208 South Africa; 3 Division of Clinical Pharmacology, Karolinska Institute, Karolinska University Hospital Huddinge, SE-141 86 Stockholm, Sweden; 4 Strathclyde Institute of Pharmacy and Biomedical Sciences, University of Strathclyde, Glasgow, G4 0RE UK; 5 Health Economics Centre, Liverpool University Management School, Chatham Street, Liverpool, UK

**Keywords:** Adverse drug reactions, Health care professionals, Pharmacovigilance, Ambulatory care, South Africa

## Abstract

**Purpose:**

Adverse drug reactions (ADRs) have an appreciable impact on patients’ health. Little is known however about ADR reporting in ambulatory care environments especially in low- and middle-income countries. Consequently, our aim was to determine knowledge, attitudes and practices (KAP) among health care professionals (HCPs) towards ADR reporting in primary health care (PHC) facilities in South Africa. The findings will be used to direct future activities.

**Methods:**

Descriptive, cross-sectional design using quantitative methodology among 8 public sector community health care centres and 40 PHC clinics in the Tshwane Health District, Gauteng Province. A self-administered questionnaire was distributed to 218 HCPs, including all key groups.

**Results:**

A total of 200 responses were received (91.7%). Although an appropriate attitude towards ADR reporting existed, the actual frequency of ADR reporting was low (16.0%). Of the respondents, 60.5% did not know how to report, where to report or when to report an ADR and 51.5% said the level of their clinical knowledge made it difficult to decide whether or not an ADR had occurred. Over 97.5% stated they should be reporting ADRs with 89% feeling that ADR reporting is a professional obligation and over 70% that ADR reporting should be compulsory. When results were combined, the overall mean score in terms of positive or preferred practices for ADR reporting was 24.6% with pharmacists having the highest scores.

**Conclusion:**

Under-reporting of ADRs with gaps in KAP was evident. There is a serious and urgent need for education and training of HCPs on ADR reporting in South Africa.

**Electronic supplementary material:**

The online version of this article (10.1007/s00228-020-02862-8) contains supplementary material, which is available to authorized users.

## Introduction

Adverse drug reactions (ADRs) are a major public health problem that causes increased mortality, morbidity and costs, including increased hospital admissions and length of stay [1–9]. Physicians, pharmacists, dentists and nurses are in a position to play a key role in pharmacovigilance programmes; however, under-reporting of ADRs is common across countries especially in low- and middle-income countries (LMICs) [10–18].

Health care professionals (HCPs), especially in LMICs, should work together to remove barriers to ADR reporting across sectors and establish effective pharmacovigilance systems [15, 17, 19–24]. This includes physicians, pharmacists and nurses in ambulatory care in LMICs including South Africa [25–29]. Such activities should result in safety signals being detected at an earlier stage, leading to better and quicker decisions about medicine use. However, this cooperation means improved notification and recording of ADRs in ambulatory care where the majority of patients receive their medicines.

ADR spontaneous reporting is currently the basic method for collecting information about adverse post-marketing risks and events [17, 30]. Spontaneous reporting systems are inexpensive and simple to operate, and form the core of the global World Health Organization (WHO) database [31]. Their strength is connected to actual reporting rates of ADRs by HCPs, recognising through appreciable under-reporting in many countries [32–37].

Many factors are associated with ADR under-reporting among HCPs, referred to as ‘the seven deadly sins’ of pharmacovigilance [38]. These include a lack of knowledge about the necessary forms, ignorance of the rules and procedures and type of events that must be reported and lack of time and inertia, as well as lack of education among all key stakeholder groups [12, 16, 17, 19, 21, 23, 24, 37–40]. In addition, currently only a limited number of African countries have formal ADR reporting systems. Countries include Morocco, South Africa, Tanzania, Tunisia, Zimbabwe, Ghana, Egypt, Nigeria, Mozambique, Uganda and Togo, all of which are full members of the WHO Programme for International Drug Monitoring [41]. Progress has been hampered by lack of training and funding [42]. However, pharmacovigilance activities should increase with 35 African countries now part of the WHO Programme for International Drug Monitoring [41, 43]. The number of ADRs reported from African countries is also growing which is encouraging, with, for instance, South Africa reporting 28,609 individual cases by the end of 2015 [43]. However, more needs to be done. Implementation of successful spontaneous reporting systems requires resources of staff and systems. Concerns in LMICs include the remote location of a number of ambulatory care clinics and/or primary healthcare (PHC) clinics and poor telecommunication services, as well as low numbers of HCPs and inadequate training [44]. The knowledge of, attitudes towards and practices of spontaneous reporting may also differ at various levels of health care systems [15, 16, 19, 24].

Concerns with the under-reporting of ADRs in South Africa led to the establishment of a Pharmacovigilance Committee within the previous Medicines Control Council (MCC) of South Africa [45], providing direction on ADR reporting [46]. The MCC has now been replaced by the South African Health Products Regulatory Authority (SAHPRA) [47, 48]. Post-marketing reporting of ADRs is a legal requirement. All serious or suspected ADRs must be reported to the regulatory authority by the medicine licence holder or applicant within 15 days of receipt of such information [46]. There are also initiatives among the provinces (regions) to promote pharmacovigilance activities to increase the number of ADR reports [49].

Between 2012 and 2017, pilot projects were rolled-out in 10 health districts in South Africa to evaluate various health system strengthening interventions focused at the PHC level [50], in preparation for the implementation of the National Health Insurance (NHI) scheme for universal healthcare including improved quality of care and services. One of these districts was the Tshwane Health District in Pretoria, delivering PHC services through community health centres (CHCs) incorporating PHC clinics as the first point of entry to healthcare services. Health status reports, including ADRs, are discussed at the Tshwane Health District Pharmaceutical and Therapeutics Committee (PTC) meetings on a quarterly basis. PTCs are now a formal requirement across sectors in South Africa [51]. Over the 18-month period prior to this study, very few ADRs were considered, which is a concern. Actual numbers were not available due to poor record-keeping.

To date, few published studies have determined which factors relate to under-reporting of ADRs among PHC facilities especially in LMICs [15, 19]. This compares to multiple studies among hospitals including South Africa where there are concerns with the lack of reporting of ADRs although this is now being addressed [1, 3–5, 14, 16, 17, 52–57].

This is a critical concern given the high prevalence of both infectious and non-communicable diseases across Africa [58–64]. In addition, there are appreciable differences in patients with HIV in sub-Saharan Africa compared with western countries, with a higher percentage being women, leading to appreciable genetic differences between the populations [63, 65]. There are also a considerable number of patients with concomitant infectious diseases, including HIV, alongside NCDs in sub-Saharan Africa, impacting on potential ADRs with patients likely to be on multiple medications [66, 67].

An overview of concerns regarding the lack of reporting of ADRs at a secondary level hospital in South Africa has recently been published alongside potential ways to address this [16, 52]. To the best of our knowledge, information about these variables among PHC facilities in South Africa has not been published. This omission is a concern given the number of patients treated at public PHC facilities in the country, coupled with ongoing initiatives to improve the care of patients with chronic diseases [68]. This study was undertaken to determine the current ADR reporting situation among PHC facilities in Tshwane Health District. The findings could be used to design and implement programmes to improve ADR reporting at PHC facilities in the province and other sites and could also be of interest to other African countries striving to improve healthcare delivery.

## Methods

### Study design and setting

A descriptive, cross-sectional design used quantitative methodology and a self-administered questionnaire. The study was conducted among all 48 PHC facilities in the Tshwane Health District (8 CHCs and 40 PHC clinics), situated in the Gauteng Province of South Africa. This district was chosen as it was a pilot area for the introduction of NHI.

In the public sector, approximately 80% of consultations at the PHC level are with a professional nurse. PHC clinics are smaller facilities, mainly staffed by nurses and sometimes a visiting physician. A pharmacist will visit clinics once a month and, at the time of the study, post-basic pharmacist assistants (PBPAs) were being introduced into clinics’ staff complement, working under the direct supervision of a pharmacist. CHCs are larger facilities than clinics, staffed by a multidisciplinary PHC team consisting of professional nurses, physicians, a pharmacist and PBPAs. Some CHCs operate 24 h per day with staff rotating.

### Study population and participants

The study population consisted of 475 HCPs (38 physicians, 317 professional nurses, 10 pharmacists and 110 PBPAs) employed at the 48 PHC facilities in the Tshwane Health District at the time of the study.

A combined sample size estimation of all HCP categories was performed on nQuery Advisor, Release 7.0, considering staff rotations, visiting staff and that all facilities are not equally staffed. It was estimated that with a combined sample size of 212 respondents, a two-sided 95% confidence interval for the percentage HCPs with satisfactory knowledge, attitude and practices would be within ± 5% of the percentage that would be calculated from the sample, assuming that 80% of the respondents had satisfactory knowledge, attitudes and practices.

Convenience sampling was employed. HCPs who were available on the day of data collection and who complied with the following inclusion criteria were approached:

- HCPs permanently employed by the Gauteng Department of Health
- Registered pharmacists, professional nurses, physicians and PBPAs
- Willingness to participate in the study
- Provision of written informed consent.

### Data collection instrument and process

A self-administered, structured questionnaire was developed based on previous practice experience among the co-authors, discussions with experts and consideration of the literature [69]. Two experts in the field of pharmacovigilance reviewed the questionnaire for content validity; after which, it was tested among 6 HCPs for feasibility. The questionnaire was subsequently revised to improve its robustness to achieve appropriate outcomes (Appendix 1 in the Supplementary Material).

Potential participants at the clinics were approached by one of the six community service pharmacists. The aim and objectives of the study were explained to them and written consent to participate was obtained (Appendices 2 and 3 in the Supplementary Material). A total of 218 questionnaires were distributed. The questionnaires were handed to participants for anonymous completion in a private room. On completion, respondents placed questionnaires in a sealed box to ensure confidentiality of responses.

### Data entry and analysis

Data were captured using Microsoft Excel™, checked for accuracy and cleaned before analysis with SAS, release 9.2, running under Microsoft Windows.

Responses were categorised according to knowledge, attitudes and practices based on the questions included in the questionnaire. Correct or preferred responses were subject to frequency counts and percentages for each item as well as for the respective HCP category.

An individual overall mean score (%) was calculated for each participant according to their knowledge, attitudes and practices, followed by an overall mean and median (%) score for each HCP category. Mean (%) scores for the different HCP categories were compared by analysis of variance (ANOVA), followed by pairwise comparisons using the *t* test. Statistical significance was set at *p* < 0.05. An overall mean score (%) with a 95% confidence interval (CI) for all participants was also calculated for knowledge, attitudes and practices.

### Ethical considerations

Ethical clearance for the study was obtained from the Medunsa Research Ethics Committee of the University of Limpopo, now Sefako Makgatho Health Sciences University, prior to the commencement of the study (MREC/H270/2013). Permission to conduct the study at the PHC facilities was obtained from the Tshwane Research Committee and the Chief Director of the Tshwane Health District. All participants provided written informed consent.

## Results

### Demographic characteristics

Two hundred of the 218 distributed questionnaires were completed, giving a response rate of 91.7%. No questionnaires were excluded from the analysis. One hundred and sixty-six (83%) respondents were female with 73.0% employed at PHC clinics and 27% at CHCs. Table 1 gives further details of respondents’ professions.

**Table 1:** Knowledge of health care professionals on adverse drug reactions (ADRs) (*n* = 200)

Item		Pharmacist (*n* = 10)	Medical practitioner (*n* = 23)	Professional nurse (*n* = 89)	Post-basic pharmacist assistant (*n* = 78)	Total (*n* = 200)
Number (%) of correct responses per health care professional category						
Understanding the term ‘adverse drug reaction’		10 (100%)	19 (82.6%)	66 (74.2%)	56 (71.8%)	151 (75.6%)
Know that ADRs must be reported		10 (100%)	18 (78.3%)	87 (97.8%)	70 (89.7%)	185 (92.5%)
Know of existence of an ADR reporting and monitoring system in district		7 (70.0%)	12 (52.1%)	58 (65.2%)	38 (48.7%)	115 (57.5%)
Know where to find the form to complete for reporting ADRs		6 (60.0%)	4 (17.4%)	33 (37%)	23 (29.5%)	66 (33.0%)
Know where the ADR reporting form must be submitted		3 (30.0%)	2 (8.7%)	7 (7.9%)	6 (7.7%)	18 (9.0%)
An event related to these items must be reported	Allopathic drugs	9 (90.0%)	15 (65.2%)	55 (61.8%)	40 (51.3%)	119 (59.5%)
	Herbal drugs	9 (90.0%)	15 (65.2%)	56 (62.9%)	41 (52.6%)	121 (60.5%)
	Traditional and complementary medicine	8 (80.0%)	15 (65.2%)	65 (73.0%)	45 (57.7%)	133 (66.5%)
	Blood products	8 (80.0%)	22 (95.7%)	76 (85.4%)	52 (66.7%)	158 (79.0%)
	Biologicals	9 (90.0%)	18 (78.3%)	64 (73.0%)	47 (60.3%)	138 (69.0%)
	Medical devices	10 (100%)	21 (91.3%)	79 (88.8%)	54 (69.2%)	164 (82.0%)
	Vaccines	8 (80.0%)	22 (95.7%)	81 (91.0%)	61 (78.2%)	172 (86.0%)
Main objectives of pharmacovigilance in the public sector	Improve patient care and safety	10 (100%)	23 (100%)	86 (96.6%)	69 (88.5%)	188 (94.0%)
	Improve public health and safety	10 (100%)	23 (100%)	84 (94.4%)	68 (87.2%)	185 (92.5%)
	Contribute to assessment of risk/benefit of medicines	10 (100%)	22 (95.7%)	87 (97.8%)	66 (84.6%)	185 (92.5%)
	Promote understanding, education and clinical training in field	9 (90.0%)	23 (100%)	84 (94.4%)	67 (85.9%)	183 (91.5%)
	Ensure effective communication of ADR reporting to public	10 (100%)	20 (87.0%)	83 (93.3%)	68 (87.2%)	181 (90.5%)
Mean and median (%) knowledge score on ADRs per health care professional category						
Mean % (standard deviation)		91.4 (12.3)	82.8 (14.7)	84.0 (17.0)	72.2 (22.0)	79.6* (19.6)
Median % (quartile 1–quartile 3)		95.2 (85.7–100)	85.7 (71.4–95.2)	90.0 (76.2–95.2)	76.2 (61.9–90.5)	85.7 (71.4–95.2)

*95% CI 76.9–82.3

### Health care professionals’ knowledge of what should be reported as ADRs

More than three-quarters of respondents understood the term ‘adverse drug reaction’ (75.6%) with 92.5% aware that ADRs must be reported (Table 1). Over 90% were also aware of the objectives of pharmacovigilance in the public sector. However, only 57.5% were aware of an ADR reporting and monitoring system in the district, only 33% where to find the forms and only 9.0% where to submit them (Table 1). Table 1 also contains data related to the need for reports for important treatment options as well as the breakdowns by specific HCP groups.

The overall mean knowledge scores for all participants, based on individual mean scores, are also presented in Table 1. Medical practitioners (82.8%; *p* = 0.0174), pharmacists (91.4%; *p* = 0.0025) and professional nurses (84.0%; *p* < 0.0001) scored significantly higher than PBPAs (72.2%).

**Fig. 1:**
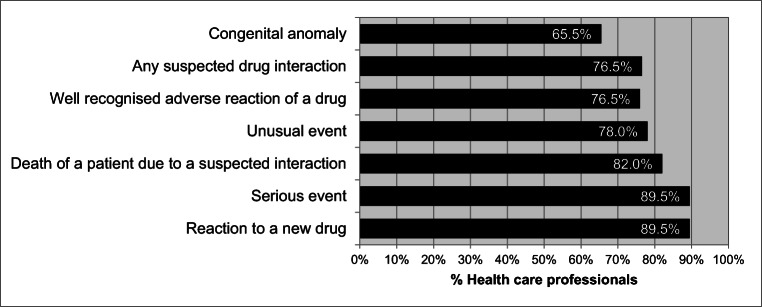
Percentage of health care professionals with knowledge of the type of events that should be reported (*n* = 200)

The respondents showed reasonably good knowledge of the type of adverse events that should be reported, ranging from 65.5% for congenital anomaly to 89.5% for reaction to a new medicine and a serious event (Fig. 1).

### Attitudes towards adverse drug reaction reporting

Table 2 contains data related to the attitudes and importance of ADR reporting. A high percentage (89.0%) agreed that reporting ADRs is a professional obligation and that there was a need for training. ADR reporting is also considered very important in everyday work (63.0%).

**Table 2:** Attitudes of health care professionals towards the reporting of adverse drug reactions (ADRs) (*n* = 200)

Item		Pharmacist (*n* = 10)	Medical practitioner (*n* = 23)	Professional nurse (*n* = 89)	Post-basic pharmacist assistant (*n* = 78)	Total positive responses (*n* = 200)
Number (%) of positive responses per health care professional category						
ADR reporting is necessary		10 (100%)	21 (91.0%)	84 (94.4%)	67 (85.9%)	182 (91.0%)
ADR reporting is a professional obligation		10 (100%)	18 (78.3%)	87 (97.8%)	63 (80.8%)	178 (89.0%)
Need for training on ADR reporting		7 (70.0%)	20 (87.0%)	78 (87.6%)	73 (93.6%)	178 (89.0%)
ADR reporting should be	Voluntary	4 (40.0%)	3 (13.0%)	5 (5.6%)	8 (10.3%)	20 (10.0%)
	Compulsory	6 (60.0%)	13 (56.5%)	76 (85.4%)	54 (69.2%)	149 (74.5%)
	Remunerated	0	2 (8.7%)	2 (2.2%)	1 (1.3%)	5 (2.5%)
Health care worker’s role	Preventing ADRs	9 (90.0%)	22 (95.7%)	83 (93.3%)	71 (91.0%)	185 (92.5%)
	Detecting ADRs	10 (100%)	23 (100%)	85 (95.5%)	64 (82.1%)	182 (91.0%)
	Managing ADRs	10 (100%)	23 (100%)	85 (95.5%)	63 (80.8%)	181 (90.5%)
	Reporting ADRs	10 (100%)	22 (95.7%)	88 (98.9%)	75 (96.2%)	195 (97.5%)
The importance of pharmacovigilance in everyday work	Very important	5 (50.0%)	13 (56.5%)	57 (64.0%)	51 (65.4%)	126 (63.0%)
	Important	5 (50.0%)	7 (30.4%)	21 (23.6%)	20 (25.6%)	53 (26.5%)
	Slightly important	0	0	0	0	0
	Not important at all	0	0	0	0	0
Mean and median (%) attitude score on ADRs per health care professional category						
Mean % (standard deviation)		73.9 (18.5)	66.7 (15.5)	68.3 (17.8)	55.2 (16.9)	63.3* (18.3)
Median % (quartile 1–quartile 3)		77.8 (66.7–83.3)	66.7 (61.1–83.3)	72.2 (55.6–83.3)	50.0 (44.4–66.7)	63.9 (50.0–77.8)

*95% CI 60.7–65.83

Individual overall attitude scores (%) were calculated for each HCP participant, based on positive or preferred responses, and then combined in group scores. The overall mean positive or applicable attitude score was 63.3% (95% CI 60.7–65.8%). The mean attitude scores of medical practitioners (66.7%; *p* = 0.0055), pharmacists (73.9%; *p* = 0.0011) and professional nurses (68.3%; *p* < 0.0001) were significantly greater than those of PBPAs (55.2%).

The major factors which were perceived to discourage ADR reporting are listed in Table 3. Nearly two-thirds of participants (60.5%) did not know how to report, where to report or when to report an ADR. Over half of the HCPs (51.5%) said that the level of their clinical knowledge made it difficult to decide whether or not an ADR had occurred.

**Table 3:** Major factors which discouraged reporting of adverse drug reactions (ADRs) (*n* = 200)

Factors discouraging ADR reporting	Number (%) of HCPs (*n* = 200)
A single unreported case may not affect the ADR data base	38 (19.0%)
Non-remuneration for reporting	47 (23.5%)
Lack of confidence to discuss the ADRs with other colleagues	56 (28.0%)
Concern that reporting may generate extra work	67 (33.5%)
Lack of time to actively look for ADRs whilst at work	79 (39.5%)
Lack of time to complete a report	88 (44.0%)
Concern that the report may be incorrect	93 (46.5%)
Level of clinical knowledge makes it difficult to decide whether or not an ADR has occurred	103 (51.5%)
Do not know how to report, where to report and when to report	121 (60.5%)

**Fig. 2:**
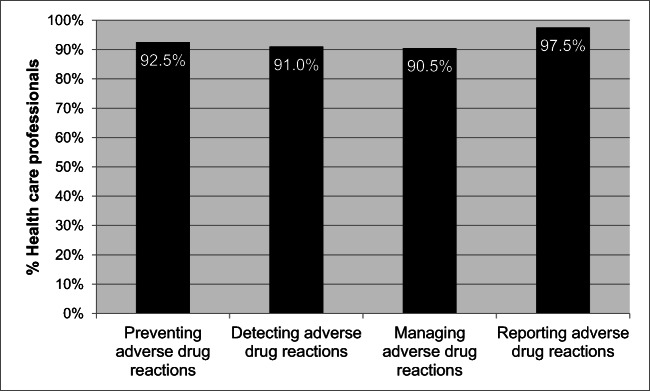
Health care professionals’ perceived roles in ADR reporting (*n* = 200)

Respondents’ perceptions of possible roles of HCPs in responding to ADRs are contained in Fig. 2. Over 97% stated that they should be reporting ADRs with over 92% stating that they should try to prevent ADRs when selecting medicines to treat their patients.

### HCP current practice of ADR reporting

Table 4 shows that only 16.0% of HCPs surveyed had ever reported a suspected ADR, although 65.0% said that ADR forms were available in their facilities and only 12.0% knew where the forms were kept.

**Table 4:** Practice of health care professionals in adverse drug reaction (ADR) reporting (*n* = 200)

Item		Pharmacist (*n* = 10)	Medical practitioner (*n* = 23)	Professional nurse (*n* = 89)	Post-basic pharmacist assistant (*n* = 78)	Total correct responses (*n* = 200)
Number (%) correct responses per health care professional category						
Have you ever reported any suspected ADR?	Yes	4 (40.0%)	6 (26.1%)	12 (13.5%)	10 (12.8%)	32 (16.0%)
Have you reported any suspected ADR to the ADR reporting and monitoring system in your district?	Yes	1 (10.0%)	3 (13.0%)	4 (4.5%)	6 (7.7%)	14 (7.0%)
Do you have the adverse reporting form available in your facility?	Yes	7 (70.0%)	10 (43.5%)	70 (78.7%)	43 (55.0%)	130 (65.0%)
Where are the ADR forms kept in your facility?	Pharmacy/managers office	3 (30.0%)	3 (13.0%)	13 (14.6%)	5 (2.5%)	19 (12.0%)
Copies of the submitted ADR forms are kept	Yes	2 (20.0%)	6 (26.1%)	37 (41.6%)	28 (35.9%)	73 (36.5%)
Copy of form attached to questionnaire	Yes	0	0	2 (2.2%)	1 (1.3%)	3 (1.5%)
Training received on ADR reporting	Yes	4 (40.0%)	4 (17.4%)	18 (20.2%)	8 (10.3%)	34 (17.0%)
Mean and median (%) practice score on ADRs per health care professional category						
Mean % (standard deviation)		33.3 (31.4)	21.7 (24.3)	27.5 (18.0)	20.9 (20.4)	24.6* (20.7)
Median % (quartile 1–quartile 3)		25.0 (0.0–66.7)	16.7 (0.0–33.3)	33.3 (16.7–33.3)	16.7 (0.0–33.0)	16.7 (8.3–33.3)

*95% CI 21.7–27.4

In contrast to the 16.0% of respondents who stated that they had reported an ADR, more than a third (36.5%) of respondents said that they kept copies of the forms they submitted, but only three could attach a copy of the completed form. This anomaly casts doubts on their understanding of these two questions. Only 17.0% of respondents indicated they had ever received training on ADR reporting, more among pharmacists than other HCPs (Table 4).

When all practice questions and statements for all HCPs were combined, the overall mean score in terms of positive or preferred practices for ADR reporting was 24.6% (95% CI 21.7–27.4%). The mean practice score for PBPAs (20.9%) was significantly lower than the mean for pharmacists (33.3%; *p* = 0.050), whilst not significantly different from the mean practice scores of medical practitioners (21.7%) and professional nurses (27.5%).

Based on the overall mean scores, pharmacists achieved the highest ranking in terms of knowledge, attitudes and practice. Although the differences were not statistically significant, these findings would not have been out of place due to pharmacists’ training focussing on medicines.

## Discussion

Whilst a positive attitude to ADR reporting existed among HCPs working at PHC facilities in our study, the actual practice of ADR reporting was poor, similar to studies in other LMICs including India [57, 69–73], Pakistan [54] and Romania [74].

Our results reflected a lack of awareness (57.5%) of HCPs about the existence of an ADR reporting system (Table 1), reflected by very few HCPs ever reporting an adverse event (16.0%) or contributed to the ADR monitoring system in the district (7.0%) (Table 4), again similar to other LMICs including secondary care facilities in South Africa [16, 17, 72, 75]. However, reporting rates in this study are appreciably lower than seen in India where 47% of respondent physicians had reported an ADR [71] and Malaysia where 51.9% of physicians and pharmacists in PHC facilities had reported an ADR in the past year [19].

Whilst the majority of HCPs surveyed (89.0%) felt that ADR reporting is a professional obligation (Table 2) similar to other countries [17, 19, 56, 71, 74, 76], they would be encouraged to report ADRs if the reaction is serious (89.5%), for a new product (89.5%) or unusual (78.0%) (Fig. 1), similar to other studies [75–77]. However, 22 (11.0%) of HCPs were unaware of the professional obligation to report ADRs (Table 2). Personal discussions and awareness programmes should help to remove misconceptions and modify attitudes so that ADR reporting becomes an integral part of clinical practice [19, 76, 78, 79]. The attitude of HCPs that a single unreported case may not affect the ADR database (19.0%) also needs to be challenged and changed. Addressing this through education may well lead to enhanced spontaneous reporting.

ADRs of herbal and traditional medicines are well known [80–83]. However, only 60.5% of HCPs considered it necessary to report events related to herbal drugs which is a concern (Table 1). This is important in South Africa and across Africa since many such remedies are currently being used [84–86] and there is appropriate legislation to address this [87]. However, this attitude may reflect a general reluctance among the population to admit to seeking traditional remedies although such practices may be common especially among rural black households in South Africa [88].

A number of factors impacted negatively on the willingness to report ADRs of which ‘Do not know how to report, where to report and when to report’ (60.5%) was the most prominent (Table 3). These findings suggest that under-reporting of ADRs is associated with gaps in knowledge, attitudes and practices, similar to other studies including hospital-based studies [17–19, 23, 72, 74, 76]. Concerns with lack of knowledge may reflect a spontaneous reporting rate of only 16.0% (Table 4), similar to other reported studies [72, 75]. Another concern was that only 12.0% of HCPs knew where the ADR forms were kept (Table 4). These joint findings again suggest a serious and urgent need for appropriate education and training, from identification to reporting, which should improve spontaneous reporting [19, 72, 76, 89–91]. We have seen the successful implementation of a pharmacist-directed improvement plan to enhance ADR reporting in secondary care in South Africa [52]. Whether a similar approach would be successful over a number of widespread PHC facilities remains to be seen. However, such approaches are being tried in other countries [19, 72, 78], and we will be following the findings with interest. In the meantime, all HCPs in South Africa should be encouraged to report suspected ADRs irrespective of the level of association with the possible cause [36]. Reporting whether known, unknown, common, uncommon, serious or mild ADR, even with established medicines, should be encouraged. Training in pharmacovigilance should also be included in the core curriculum of all HCPs following government initiatives [47], helped by the introduction of a new reporting form [48, 68]. Provision is also being made for a mobile electronic version (App), which would include an acknowledgement of a receipt sent to the reporter to address concerns. These initiatives will be investigated in future studies for their impact.

In addition, SAHPRA is in the process of strengthening its vigilance and post-marketing surveillance programme, including the development of a communication strategy to support improved external stakeholder interactions and relations. Providing relevant and user-friendly feedback to stakeholders, particularly health professionals and the public, should start to address previous concerns [47].

We are aware of a number of limitations with this study such as including only one district and an unequal distribution of participants from the different HCP categories. For instance, there was low participation (28%) from nurses. We are not sure why but possible reasons could include being reluctant to participate, being too busy or being on night duty at the 24-h CHCs. However, all pharmacists as well as a high percentage of PBPAs (71%) and physicians (60%) took part. In addition, qualitative research methodologies would have provided a more in-depth understanding. Future research is planned to address these concerns. Despite these limitations, we believe our findings are robust and provide direction for the future as the authorities strive to improve the use of medicines in ambulatory care.

## Conclusion

Our findings strongly suggest that under-reporting of ADRs is associated with gaps in knowledge, attitudes and practices among ambulatory care HCPs in South Africa. Consequently, there is a great need to create awareness about ADRs and to promote the reporting of ADRs among HCPs. This is especially important given the rising burden of non-communicable diseases in South Africa along with infectious diseases. Training sessions should help, augmented by structured surveillance and electronic methods of data handling, analysis and the generation of ADR reports. In addition, ADR reporting must be seen as an integral part of undergraduate training and the clinical activities of all ambulatory care HCPs. We will be monitoring this process in the future.

## Electronic supplementary material

ESM 1 (DOCX 69 kb)

## References

[1] Mouton JP, Mehta U, Parrish AG, Wilson DPK, Stewart A, Njuguna CW, Kramer N, Maartens G, Blockman M, Cohen K (2015). Mortality from adverse drug reactions in adult medical inpatients at four hospitals in South Africa: a cross-sectional survey. Br J Clin Pharmacol.

[2] Mouton JP, Njuguna C, Kramer N, Stewart A, Mehta U, Blockman M, Fortuin-de Smidt M, de Waal R, Parrish AG, Wilson DP, Igumbor EU, Aynalem G, Dheda M, Maartens G, Cohen K (2016). Adverse drug reactions causing admission to medical wards: a cross-sectional survey at 4 hospitals in South Africa. Medicine..

[3] Benard-Laribiere A, Miremont-Salame G, Perault-Pochat MC, Noize P, Haramburu F (2015). Incidence of hospital admissions due to adverse drug reactions in France: the EMIR study. Fundam Clin Pharmacol.

[4] Tumwikirize WA, Ogwal-Okeng JW, Vernby A, Anokbonggo WW, Gustafsson LL, Lundborg SC (2011). Adverse drug reactions in patients admitted on internal medicine wards in a district and regional hospital in Uganda. Afr Health Sci.

[5] Martins AC, Giordani F, Rozenfeld S (2014). Adverse drug events among adult inpatients: a meta-analysis of observational studies. J Clin Pharm Ther.

[6] Davies EC, Green CF, Taylor S, Williamson PR, Mottram DR, Pirmohamed M (2009). Adverse drug reactions in hospital in-patients: a prospective analysis of 3695 patient-episodes. PLoS One.

[7] Pirmohamed M, James S, Meakin S, Green C, Scott AK, Walley TJ (2004) Adverse drug reactions as cause of admission to hospital: prospective analysis of 18 820 patients. Br Med J 329 10.1136/bmj.329.7456.15 PMC443443 15231615

[8] Hug BL, Keohane C, Seger DL, Yoon C, Bates DW (2012). The costs of adverse drug events in community hospitals. Jt Comm J Qual Patient Saf.

[9] Bouvy JC, De Bruin ML, Koopmanschap MA (2015). Epidemiology of adverse drug reactions in Europe: a review of recent observational studies. Drug Saf.

[10] Ogar CK, Abiola A, Yuah D, Ibrahim A, Oreagba IA, Amadi EC, Adeyeye MC, Oshikoya KA (2019). A retrospective review of serious adverse drug reaction reports in the Nigerian VigiFlow database from September 2004 to December 2016. Pharmaceut Med.

[11] Oumar AA, Dakouo M, Tchibozo A, Maiga M, Landoure G, Abdi-Bogoreh R (2019). Antiretroviral-induced adverse drug reactions in HIV-infected patients in Mali: a resource-limited setting experience. Int J Basic Clin Pharmacol.

[12] Adenuga BA, Kibuule D, Rennie TW (2019) Optimizing spontaneous adverse drug reaction reporting in public healthcare setting in Namibia. Basic Clin Pharmacol Toxicol 10.1111/bcpt.13325 31520574

[13] Jones J, Mudaly V, Voget J, Naledi T, Maartens G, Cohen K (2019). Adverse drug reactions in South African patients receiving bedaquiline-containing tuberculosis treatment: an evaluation of spontaneously reported cases. BMC Infect Dis.

[14] Katusiime B, Semakula D, Lubinga SJ (2015). Adverse drug reaction reporting among health care workers at Mulago National Referral and teaching hospital in Uganda. Afr Health Sci.

[15] Emeka P, Badger-Emeka L. A study on the knowledge and barriers towards ADRs reporting among community pharmacists in Enugu and Nsukka are-as, South-Eastern Nigeria 2017. 1–6 p

[16] Terblanche A, Meyer JC, Godman B, Summers RS (2017). Knowledge, attitudes and perspective on adverse drug reaction reporting in a public sector hospital in South Africa: baseline analysis. Hospital practice.

[17] Amrain MBF (2014). Knowledge, perception, practices and barriers of healthcare professionals in Bosnia and Herzegovina towards adverse drug reaction reporting. J Health Sci.

[18] Peymani P, Tabrizi R, Afifi S, Namazi S, Heydari ST, Shirazi MK, Nouraei H, Sadeghi E, Lankarani KB, Maharlouei N (2016). Knowledge, attitude and practice of general practitioners towards adverse drug reaction reporting in South of Iran, Shiraz (Pharmacoepidemiology report). Int J Risk Saf Med.

[19] Tew MM, Teoh BC, Mohd Baidi AS, Saw HL (2016) Assessment of knowledge, attitude and practices of adverse drug reaction reporting among doctors and pharmacists in primary healthcare. Adv Pharmacoepidemiol drug Saf 5:206. Available at URL: https://www.longdom.org/open-access/assessment-of-knowledge-attitude-and-practices-of-adverse-drugreaction-reporting-among-doctors-and-pharmacists-in-primaryhealthcar-2167-1052-1000206.pdf

[20] Backstrom M, Mjorndal T, Dahlqvist R (2002). Spontaneous reporting of adverse drug reactions by nurses. Pharmacoepidemiol Drug Saf.

[21] Backstrom M, Mjorndal T, Dahlqvist R, Nordkvist-Olsson T (2000). Attitudes to reporting adverse drug reactions in northern Sweden. Eur J Clin Pharmacol.

[22] Baek HJ, Cho YS, Kim KS, Lee J, Kang HR, Suh DI (2016). Multidisciplinary approach to improve spontaneous ADR reporting in the pediatric outpatient setting: a single-institute experience in Korea. Springerplus..

[23] Le TT, Nguyen TTH, Nguyen C, Tran NH, Tran LA, Nguyen TB (2020). Factors associated with spontaneous adverse drug reaction reporting among healthcare professionals in Vietnam. J Clin Pharm Ther.

[24] Mulchandani R, Kakkar AK (2019). Reporting of adverse drug reactions in India: a review of the current scenario, obstacles and possible solutions. Int J Risk Saf Med.

[25] Schellack N, Meyer JC, Gous AGS, Winters C (2011) Part II. GARP: health and economic context. S Afr Med J 11(8):558–561 Available at URL: http://www.samj.org.za/index.php/samj/article/view/5058/3365 21920133

[26] Rezal RS, Hassali MA, Alrasheedy AA, Saleem F, Yusof FA, Kamal M, Mohd Din R, Godman B (2015). Prescribing patterns for upper respiratory tract infections: a prescription-review of primary care practice in Kedah, Malaysia, and the implications. Expert Rev Anti-Infect Ther.

[27] Matsitse TB, Helberg E, Meyer JC, Godman B, Massele A, Schellack N (2017) Compliance to the primary health care treatment guidelines and the essential medicines list in the management of sexually transmitted infections in correctional centres in South Africa: findings and implications. Expert Rev Anti-Infect Ther 10.1080/14787210.2017.1382354 28922959

[28] Onoya D, Hirasen K, van den Berg L, Miot J, Long LC, Fox MP (2018). Adverse drug reactions among patients initiating second-line antiretroviral therapy in South Africa. Drug Saf.

[29] Rampamba EM, Meyer JC, Helberg EA, Godman B (2019). Empowering hypertensive patients in South Africa to improve their disease management: a pharmacist-led intervention. J Res Pharm Pract.

[30] Pacurariu AC, Straus SM, Trifiro G, Schuemie MJ, Gini R, Herings R (2015). Useful interplay between spontaneous ADR reports and electronic healthcare records in signal detection. Drug Saf.

[31] UMC. Uppsala Monitoring Centre. ANNUAL REPORT July 2015–June 2016. Available at URL: https://www.who-umc.org/media/3081/umc-annual-report-final-version_small.pdf [

[32] Vessal G, Mardani Z, Mollai M (2009). Knowledge, attitudes, and perceptions of pharmacists to adverse drug reaction reporting in Iran. Pharm World Sci.

[33] Tandon VR, Mahajan V, Khajuria V, Gillani Z (2015). Under-reporting of adverse drug reactions: a challenge for pharmacovigilance in India. Indian J Pharm.

[34] Palleria C, Leporini C, Chimirri S, Marrazzo G, Sacchetta S, Bruno L, Lista RM, Staltari O, Scuteri A, Scicchitano F, Russo E (2013). Limitations and obstacles of the spontaneous adverse drugs reactions reporting: two “challenging” case reports. J Pharmacol Pharmacother.

[35] Ribeiro-Vaz I, Santos C, da Costa-Pereira A, Cruz-Correia R (2012). Promoting spontaneous adverse drug reaction reporting in hospitals using a hyperlink to the online reporting form: an ecological study in Portugal. Drug Saf.

[36] Mehta U, Kalk E, Boulle A, Nkambule P, Gouws J, Rees H, Cohen K (2017). Pharmacovigilance: a public health priority for South Africa. South African Health Rev.

[37] Shamim S, Sharib SM, Malhi SM, Muntaha SU, Raza H, Ata S (2016). Adverse drug reactions (ADRS) reporting: awareness and reasons of under-reporting among health care professionals, a challenge for pharmacists. Springerplus.

[38] Inman WH (1996). Attitudes to adverse drug reaction reporting. Br J Clin Pharmacol.

[39] Fadare J, Enwere O, Afolabi O, Chedi B, Musa A (2011) Knowledge, attitude and practice of adverse drug reaction reporting among healthcare workers in a tertiary centre in Northern Nigeria. Tropical Journal of Pharmaceutical Research 10 (3): 235-242

[40] Adisa R, Adeniyi OR, Fakeye TO (2019). Knowledge, awareness, perception and reporting of experienced adverse drug reactions among outpatients in Nigeria. Int J Clin Pharm.

[41] World Health Organisation. The WHO Programme for International Drug Monitoring. Available at URL: http://www.who.int/medicines/areas/quality_safety/safety_efficacy/National_PV_Centres_Map/en/

[42] Olsson S, Pal SN, Stergachis A, Couper M (2010). Pharmacovigilance activities in 55 low- and middle-income countries: a questionnaire-based analysis. Drug Saf.

[43] Ampadu HH, Hoekman J, de Bruin ML, Pal SN, Olsson S, Sartori D, Leufkens HG, Dodoo AN (2016). Adverse drug reaction reporting in Africa and a comparison of individual case safety report characteristics between Africa and the rest of the world: analyses of spontaneous reports in VigiBase®. Drug Saf.

[44] Sevene E, Mariano A, Mehta U, Machai M, Dodoo A, Vilardell D, Patel S, Barnes K, Carné X (2008). Spontaneous adverse drug reaction reporting in rural districts of Mozambique. Drug Saf.

[45] MCC. Medicines Control Council South Africa. Available at URL: http://www.mccza.com/about/

[46] MCC. Medicines Control Council and National Department of Health. Reporting Adverse Drug Reactions in South Africa. Available at URL: http://docplayer.net/37893933-Medicines-control-council.html

[47] SOUTH AFRICAN HEALTH PRODUCTS REGULATORY AUTHORITY (SAHPRA): STRATEGIC PLAN FOR THE FISCAL YEARS 2018/19–2022/23. 2018. Available at URL: https://www.sahpra.org.za/documents/30142a56SAHPRAAPP2019.pdf

[48] SOUTH AFRICAN HEALTH PRODUCTS REGULATORY AUTHORITY (SAHPRA): ADVERSE DRUG REACTIONS & QUALITY PROBLEM REPORTING FORM. 2017. Available at URL: https://www.sahpra.org.za/documents/12e54dcaADRForms.pdf

[49] Gauteng Province. GAUTENG PHARMACOVIGILANCE BULLETIN. April 2017. Available at URL: file:///C:/Users/mail/Downloads/PV%20Bulletin%2020%20April.pdf

[50] National Department of Health. Evaluation of Phase 1 implementation of interventions in the National Health Insurance (NHI) pilot districts in South Africa - Evaluation Report Final. NDOH10/2017-2018. July 2019. Available at URL: https://www.hst.org.za/publications/NonHST%20Publications/nhi_evaluation_report_final_14%2007%202019.pdf

[51] Matlala M, Gous AG, Godman B, Meyer JC (2017). Structure and activities of pharmacy and therapeutics committees among public hospitals in South Africa; findings and implications. Expert Rev Clin Pharmacol.

[52] Terblanche A, Meyer JC, Godman B, Summers RS (2018). Impact of a pharmacist-driven pharmacovigilance system in a secondary hospital in the Gauteng Province of South Africa. Hospital practice.

[53] Mouton JP, Fortuin-de Smidt MC, Jobanputra N, Mehta U, Stewart A, de Waal R, Technau KG, Argent A, Kroon M, Scott C, Cohen K (2020). Serious adverse drug reactions at two children’s hospitals in South Africa. BMC Pediatr.

[54] Nisa ZU, Zafar A, Sher F (2018). Assessment of knowledge, attitude and practice of adverse drug reaction reporting among healthcare professionals in secondary and tertiary hospitals in the capital of Pakistan. Saudi Pharm J.

[55] Chikowe I, Domingo M, Mwakaswaya V, Parveen S, Mafuta C, Kampira E (2019). Adverse drug reactions experienced by out-patients taking chlorpromazine or haloperidol at Zomba Mental Hospital, Malawi. BMC Research notes.

[56] Alsaleh FM, Lemay J, Al Dhafeeri RR, AlAjmi S, Abahussain EA, Bayoud T (2017). Adverse drug reaction reporting among physicians working in private and government hospitals in Kuwait. Saudi Pharm J.

[57] Desai CK, Iyer G, Panchal J, Shah S, Dikshit RK (2011). An evaluation of knowledge, attitude, and practice of adverse drug reaction reporting among prescribers at a tertiary care hospital. Perspect Clin Res.

[58] Irazola VE, Gutierrez L, Bloomfield G, Carrillo-Larco RM, Dorairaj P, Gaziano T, Levitt NS, Miranda JJ, Ortiz AB, Steyn K, Wu Y, Xavier D, Yan LL, He J, Rubinstein A (2016). Hypertension prevalence, awareness, treatment, and control in selected LMIC communities: results from the NHLBI/UHG network of centers of excellence for chronic diseases. Glob Heart.

[59] Nashilongo MM, Singu B, Kalemeera F, Mubita M, Naikaku E, Baker A, Ferrario A, Godman B, Achieng L, Kibuule D (2017). Assessing adherence to antihypertensive therapy in primary health care in Namibia: findings and implications. Cardiovasc Drugs Ther.

[60] Wanyiri JW, Kanyi H, Maina S, Wang DE, Ngugi P, O’Connor R, Kamau T, Waithera T, Kimani G, Wamae CN, Mwamburi M, Ward HD (2013). Infectious diarrhoea in antiretroviral therapy-naïve HIV/AIDS patients in Kenya. Trans R Soc Trop Med Hyg.

[61] Kayima J, Wanyenze RK, Katamba A, Leontsini E, Nuwaha F (2013). Hypertension awareness, treatment and control in Africa: a systematic review. BMC Cardiovasc Disord.

[62] Cois A, Day C (2015). Obesity trends and risk factors in the South African adult population. BMC obesity.

[63] Gaida R, Truter I, Grobler C, Kotze T, Godman B (2016). A review of trials investigating efavirenz-induced neuropsychiatric side effects and the implications. Expert Rev Anti-Infect Ther.

[64] Rampamba EM, Meyer JC, Godman B, Kurdi A, Helberg E (2018). Evaluation of antihypertensive adherence and its determinants at primary healthcare facilities in rural South Africa. J Comp Eff Res.

[65] Kalemeera F CM, Mubita M, Kibuule D, Naikaku E, Massele M et al. (2017) The potential effect of using the Cockcroft-Gault method on tenofovir-associated renal impairment reports and on clinical decisions regarding tenofovir use in individual patients: implications for the future. Jn Infect Dis Preve Med. 5(3)

[66] Oni T, Berkowitz N, Kubjane M, Goliath R, Levitt NS, Wilkinson RJ (2017). Trilateral overlap of tuberculosis, diabetes and HIV-1 in a high-burden African setting: implications for TB control. Eur Respir J.

[67] Rankgoane-Pono G, Tshikuka JG, Magafu MGMD, Masupe T, Molefi M, Hamda SG, Setlhare V, Tapera R, Mbongwe B (2018). Incidence of diabetes mellitus-related comorbidities among patients attending two major HIV clinics in Botswana: a 12-year retrospective cohort study. BMC research notes.

[68] Meyer JC, Schellack N, Stokes J, Lancaster R, Zeeman H, Defty D (2017). Ongoing initiatives to improve the quality and efficiency of medicine use within the public healthcare system in South Africa. A Preliminary Study Frontiers in Pharmacology.

[69] Gupta P, Udupa A Adverse drug reaction reporting and pharmacovigilance: knowledge, attitudes and perceptions among resident doctors. J Pharm Sci Res 3:1064–1069 Available at URL: https://pdfs.semanticscholar.org/eb0f/02a269dccf0c277211ca86f70d66f6cf0ddc.pdf?_ga=2.251938121.1306576520.533844085-220803592.533844085

[70] Kharkar M, Bowalekar S (2012). Knowledge, attitude and perception/practices (KAP) of medical practitioners in India towards adverse drug reaction (ADR) reporting. Perspect Clin Res.

[71] Kunnoor NSS, Lohit K (2017). Perception of doctors towards adverse drug reaction (ADR) reporting: a cross sectional survey using a validated questionnaire. Int J Basic Clin Pharmacol Ther.

[72] Bhagavathula AS, Elnour AA, Jamshed SQ, Shehab A (2016). Health professionals’ knowledge, attitudes and practices about pharmacovigilance in India: a systematic review and meta-analysis. PLoS One.

[73] Ramesh M, Parthasarathi G. Adverse drug reactions reporting: attitudes and perceptions of medical practitioners. Asian J Pharm Clin Res. 2009; 2: 10–14.Available at URL: https://pdfs.semanticscholar.org/ab31/397db8fca885f58fbc23e1c8bb2ba59019f7.pdf

[74] Paveliu MS, Bengea-Luculescu S, Toma M, Paveliu SF (2013). Perception on adverse drug reaction reporting by physicians working in southern Romania. Maedica..

[75] Li Q, Zhang SM, Chen HT, Fang SP, Yu X, Liu D, Shi LY, Zeng FD (2004). Awareness and attitudes of healthcare professionals in Wuhan, China to the reporting of adverse drug reactions. Chin Med J.

[76] Rabiu A, Simbak N, Haque M (2014) A systematic review of knowledge, attitude and practice on adverse drug reactions and pharmacovigilance among doctors. 117–27 p

[77] Belton KJ, Lewis SC, Payne S, Rawlins MD, Wood SM (1995). Attitudinal survey of adverse drug reaction reporting by medical practitioners in the United Kingdom. Br J Clin Pharmacol.

[78] Suke SG, Kosta P, Negi H (2015). Role of pharmacovigilance in India: an overview. Online J Public Health Inform.

[79] Khan SA, Goyal C, Chandel N, Rafi M (2013). Knowledge, attitudes, and practice of doctors to adverse drug reaction reporting in a teaching hospital in India: an observational study. J Nat Sci Biol Med.

[80] Bielory L, Russin J, Zuckerman GB (2004). Clinical efficacy, mechanisms of action, and adverse effects of complementary and alternative medicine therapies for asthma. Allergy Asthma Proc.

[81] Ernst E (2002). The risk-benefit profile of commonly used herbal therapies: ginkgo, St. John’s wort, ginseng, echinacea, saw palmetto, and kava. Ann Intern Med.

[82] Bruno LO, Simoes RS, de Jesus SM, Girao M, Grundmann O (2018). Pregnancy and herbal medicines: an unnecessary risk for women’s health-a narrative review. Phytother Res.

[83] Meincke R, Pokladnikova J, Straznicka J, Meyboom RHB, Niedrig D, Russmann S, Jahodar L (2017). Allergy-like immediate reactions with herbal medicines in children: a retrospective study using data from VigiBase((R)). Pediatr Allergy Immunol.

[84] Kamsu-Foguem B, Foguem C (2014). Adverse drug reactions in some African herbal medicine: literature review and stakeholders’ interview. Integr Med Res.

[85] Maroyi A (2016). Treatment of diarrhoea using traditional medicines: contemporary research in South Africa and Zimbabwe. Afr J Tradit Complement Altern Med.

[86] Smith P. (2003) The use of herbal OTC products in South Africa. C M E l . 2 1 (2): 89–95

[87] Street RA (2016). Unpacking the new proposed regulations for South African traditional health practitioners. S Afr Med J.

[88] Semenya SS, Potgieter MJ (2014). Bapedi traditional healers in the Limpopo Province, South Africa: their socio-cultural profile and traditional healing practice. J Ethnobiol Ethnomed.

[89] Lopez-Gonzalez E, Herdeiro MT, Pineiro-Lamas M, Figueiras A (2015). Effect of an educational intervention to improve adverse drug reaction reporting in physicians: a cluster randomized controlled trial. Drug Saf.

[90] Sabblah GT, Akweongo P, Darko D, Dodoo ANO, Sulley AM (2014). Adverse drug reaction reporting by doctors in a developing country: a case study from Ghana. Ghana Med J.

[91] Khalili H, Mohebbi N, Hendoiee N, Keshtkar A-A, Dashti-Khavidaki S (2012). Improvement of knowledge, attitude and perception of healthcare workers about ADR, a pre- and post-clinical pharmacists’ interventional study. BMJ Open.

