# Supplementary material for: Knowledge, attitudes and practices of health care professionals towards adverse drug reaction reporting in public sector primary health care facilities in a South African district
Source: Eur J Clin Pharmacol. 2020 Apr 15;76(7):991–1001. doi: 10.1007/s00228-020-02862-8 (PMC7306046; doi:10.1007/s00228-020-02862-8)
Supplement: Supplementary file 1 — (DOCX 69 kb) [file 228_2020_2862_MOESM1_ESM.docx]

APPENDICES

Appendix 1: Study information leaflet

**Knowledge, Attitudes and Practices of Health Care Professionals towards Adverse Drug Reaction Reporting in Public Sector Primary Health Care Facilities in the Tshwane District**

Please read this information about the study and feel free to ask any questions should you need any clarity before deciding to take part in this study.

I am a pharmacist an MPharm student from the University of Limpopo, Medunsa Campus. For the purposes of my master’s degree, I am conducting a study at all Public Sector Primary Health Care facilities in the Tshwane District to find out what the knowledge, attitudes and practices are of the health care workers in these facilities about pharmacovigilance.

A questionnaire will be used to collect the data. The questionnaire will be completed anonymously, in a private area and will take approximately 30 minutes to complete. You should ask the data collector if any of the questions included in the questionnaire are not clear.

At any time you are free to let us know if you no longer wish to participate. Your name will not be recorded and all personal information about you will be kept confidential during and after the study.

The study has been approved by the University of Limpopo, Medunsa Campus Research Ethics Committee, by the Chief Director of the Tshwane District and by the facility manager of each facility where the research will be conducted.

If you agree to participate in the study, you will be required to sign a consent form to indicate your willingness to participate. We will be very thankful if you will be prepared to take part in this study.

Please feel free to contact myself on 071 679 0059 or my supervisor, Dr JC Meyer on 012-521 4741/5, if you have any further questions regarding this study.

Kind regards

**Michelle Haines**
Researcher / MPharm student

Appendix 2: Written Consent Form

**UNIVERSITY OF LIMPOPO (Medunsa Campus) ENGLISH CONSENT FORM**

Statement concerning participation in a Research Project

**Knowledge, Attitudes and Practices of Health Care Professionals towards Adverse Drug Reaction Reporting in Public Sector Primary Health Care Facilities
in the Tshwane District**

The researcher explained the aims and objectives to me and I was provided the opportunity to ask questions and given adequate time to rethink the issue. The aim and objectives of the study are sufficiently clear to me. I have not been pressurized to participate in any way.

I understand that participation in this study is completely voluntary and that I may withdraw from it at any time without supplying reasons.

I know that this study has been approved by the Medunsa Research Ethics Committee (MREC), University of Limpopo (Medunsa Campus) / Department of Health / Tshwane District. I am fully aware that the results of this Study will be used for scientific purposes and may be published. I agree to this, provided my privacy is guaranteed.

I hereby give consent to participate in this Study.

____________________________ ___________________

Name of participant Signature of participant

Appendix 3: Questionnaire

**Knowledge, Attitudes and Practices of Health Care Professionals towards Adverse Drug Reaction Reporting in Public Sector Primary Health Care Facilities
in the Tshwane District**

Date:_____________ Data Collector: _________________Study ID: ____________

Please tick the appropriate box and/or give the necessary information in the space provided.

Facility: CHC PHC

Qualification: Pharmacist Medical Practitioner Professional nurse

Gender: Male Female

Age range: ≤ 20 21-30 31-40

41-50 51-60 ≥61

Race: Black Coloured

Asian White

| **Questions** | | | | | | | **Office use*** |
| --- | --- | --- | --- | --- | --- | --- | --- |
| Please explain what you understand under the term adverse drug reaction. | | | | | | | K1 |
| Are you aware that adverse drug reactions must be reported? | | Yes (1) | | No (2) | | | K1 |
| Have you ever reported any suspected adverse drug reactions? | | Yes (1) | | No (2) | | | P1 |
| If yes, when did you report and how often? | | | | | | | D |
| Are you aware of the existence of an adverse drug reaction (ADR) reporting and monitoring system in your District? | | Yes (1) | | No (2) | | | K1 |
| Have you reported any suspected adverse drug reactions to the ADR reporting and monitoring system in your District? | | Yes (1) | | No (2) | | | P1 |
| What form must you use when reporting an adverse drug reaction? | | | | | | | K1 |
| Do you have the adverse reporting form available in your facility? | | Yes (1) | | No (2) | | | P1 |
| Where are the ADR forms kept in your facility? | | | | | | | P1 |
| Attach a copy of the form | | | | | | | P1 |
| Where must the adverse drug reaction form be submitted to when an adverse drug reaction is reported? | | | | | | | K1 |
| Do you keep copies of the ADR forms you submit?  If yes, please attach a copy of a form which you have submitted. | | Yes (1) | | No (2) | | | P1 |
| Do you think that ADR reporting is necessary? | | Yes (1) | | No (2) | | | A1 |
| Do you think that reporting ADRs is a professional obligation? | | Yes (1) | | No (2) | | | A1 |
| How many ADRs have been reported altogether at your facility over the past year? State number. | | | | | | | D |
| Have you ever received training on adverse drug reaction reporting? | | Yes (1) | | No (2) | | | P1 |
| If YES, when and where did you receive training on adverse drug reaction reporting? | | | | | | | D |
| If not, would you like to receive training on adverse drug reaction reporting? | | Yes (1) | | No (2) | | | A1 |
| Did you study pharmacovigilance in your undergraduate study? | | Yes (1) | | No (2) | | | D |
| If yes please stipulate at which institution you studied. | | | | | | | D |
| Adverse drug reaction reporting should be? Please tick the relevant box | Voluntary (1) | | | |  | | A1 & A2 |
|  | Compulsory (2) | | | |  | |  |
|  | Remunerated (3) | | | |  | |  |
| What kind of event will you be inclined to report? | **Yes  (1)** | **No  (2)** | **I don’t know (3)** | | | |  |
| 1. Reaction to a new drug |  |  |  | | | | K1 |
| 1. Serious event |  |  |  | | | | K1 |
| 1. Unusual event |  |  |  | | | | K1 |
| 1. Well recognised adverse reaction of a drug |  |  |  | | | | K1 |
| 1. Any suspected drug interaction |  |  |  | | | | K1 |
| 1. Death of patient due to a suspected interaction |  |  |  | | | | K1 |
| 1. Congenital anomaly |  |  |  | | | | K1 |
| 1. Other events. Please specify. | | | | | | | K1 |
| Must an event related to the following be reported? | **Yes  (1)** | **No  (2)** | **I don’t know (3)** | | | |  |
| 1. Allopathic drugs |  |  |  | | | | K1 |
| 1. Herbal drugs |  |  |  | | | | K1 |
| 1. Traditional and complementary medicine |  |  |  | | | | K1 |
| 1. Blood products |  |  |  | | | | K1 |
| 1. Biological |  |  |  | | | | K1 |
| 1. Medical devices |  |  |  | | | | K1 |
| 1. Vaccines |  |  |  | | | | K1 |
| 1. Other events. Please specify. | | | | | | | K1 |
| What will be the factors that will discourage you to report? | **Yes  (1)** | **No  (2)** | **I don’t know (3)** | | | |  |
| 1. Concern that the report may be wrong. |  |  |  | | | | A2 |
| 1. Do not know how to report, where to report and when to report. |  |  |  | | | | A2 |
| 1. Lack of time to complete a report. |  |  |  | | | | A2 |
| 1. A single unreported case may not affect the ADR database |  |  |  | | | | A2 |
| 1. Non-remuneration for reporting. |  |  |  | | | | A2 |
| 1. Concern that reporting may generate extra work. |  |  |  | | | | A2 |
| 1. Lack of time to actively look for ADRs while at work. |  |  |  | | | | A2 |
| 1. Level of clinical knowledge makes it difficult to decide whether or not an ADR has occurred. |  |  |  | | | | A2 |
| 1. Lack of confidence to discuss the ADR with other colleagues. |  |  |  | | | | A2 |
| 1. Other factors. Please specify. | | | | | | | A |
| Do you think as a health care worker you have a role to play in the following? | **Yes  (1)** | **No  (2)** | **I don’t know (3)** | | | |  |
| 1. Preventing adverse drug reactions. |  |  |  | | | | A1 |
| 1. Detecting adverse drug reactions. |  |  |  | | | | A1 |
| 1. Managing adverse drug reactions. |  |  |  | | | | A1 |
| 1. Reporting adverse drug reactions. |  |  |  | | | | A1 |
| Within the public sector, the main objectives of pharmacovigilance are as follows: | **Yes  (1)** | **No  (2)** | **I don’t know (3)** | | | |  |
| 1. Improve patient care and safety |  |  |  | | | | K1 |
| 1. Improve public health and safety |  |  |  | | | | K1 |
| 1. Contribute to the assessment of risk / benefit of the medicine |  |  |  | | | | K1 |
| 1. Promote understanding, education and clinical training in this field |  |  |  | | | | K1 |
| 1. Ensure effective communication of adverse drug reaction reporting to the public |  |  |  | | | | K1 |
| 1. Other objectives. Please specify. | | | | | | | K1 |
| How do you rate the importance of pharmacovigilance in your everyday work? | Very important (1) | | | | |  | A1 & A2 |
|  | Important (2) | | | | |  |  |
|  | Slightly important (3) | | | | |  |  |
|  | Not important at all (4) | | | | |  |  |

*Office use and not for printing: K = Knowledge; A = Attitudes; P = Practices; D = Descriptive

**Thank you for your participation!**
